# Clinical features and therapeutic options in non‐small cell lung cancer patients with concomitant mutations of *EGFR*, *ALK*, *ROS1*, *KRAS* or *BRAF*

**DOI:** 10.1002/cam4.2183

**Published:** 2019-04-24

**Authors:** Xibin Zhuang, Chao Zhao, Jiayu Li, Chunxia Su, Xiaoxia Chen, Shengxiang Ren, Xuefei Li, Caicun Zhou

**Affiliations:** ^1^ Department of Respiratory Medicine Quanzhou First Hospital Quanzhou China; ^2^ Department of Lung Cancer and Immunology Shanghai Pulmonary Hospital, Tongji University Shanghai China; ^3^ Department of Medical Oncology Shanghai Pulmonary Hospital, Tongji University Shanghai China

**Keywords:** ALK, concomitant mutations, EGFR, NSCLC, ROS1

## Abstract

**Background:**

Although oncogenic driver mutations were thought to be mutually exclusive in non‐small cell lung cancer (NSCLC), certain tumors harbor co‐occurring mutations and represent a rare molecular subtype. The evaluation of the clinical features and therapeutic response associated with this NSCLC subtype will be vital for understanding the heterogeneity of treatment response and improving the management of these patients.

**Methods:**

This retrospective study included 3774 samples from patients diagnosed with NSCLC. All samples were screened for *EGFR*, *ALK*, *ROS1*, *KRAS*, and *BRAF* mutation using the amplification‐refractory mutation system. The relationship between concomitant driver mutations and clinicopathologic characteristics, and patient clinical outcomes were evaluated.

**Results:**

Sixty‐three (1.7%) samples had more than one driver gene mutation. Among these, 43 were coalterations with an *EGFR* mutation, 20 with an *ALK* rearrangement, and eight with an *ROS1* rearrangement. Except for *ROS1* concomitant mutations that were more frequent in male patients (87.5%, *P* = 0.020), the clinicopathological features of the concomitant mutation patients were not significantly different from those harboring a single *EGFR*, *ALK*, or *ROS1* mutation. Furthermore, first‐line EGFR‐TKI treatment did not significantly improve the progression‐free survival (PFS) of patients harboring *EGFR* concomitant mutation, compared to patients harboring a single *EGFR* mutation. However, for *EGFR* concomitant mutation patients, TKI therapy was more effective than chemotherapy (median PFS of 10.8 vs 5.2 months, *P* = 0.023). Lastly, *KRAS* mutations did not influence the EGFR‐TKI therapy treatment effect.

**Conclusion:**

In this study, concomitant mutations were found in 1.7% of the NSCLC. EGFR‐TKI therapy was more effective than chemotherapy for patients harboring *EGFR* concomitant mutation, and *ROS1* concomitant mutations were more frequent in male patients. For patients harboring coalterations with an *ALK* or *ROS1* rearrangement, we should be cautious when considering the therapeutic options.

## 1 INTRODUCTION

Tyrosine kinase inhibitors (TKIs) targeting epidermal growth factor receptor (*EGFR*), anaplastic lymphoma kinase (*ALK*), and c‐ros oncogene 1 (*ROS1*) have been established as efficient cancer treatments.^1^, ^2^, ^3^, ^4^, ^5^ However, concomitant mutations in these driver genes, as well as in the *KRAS* and *BRAF* oncogenes, have been reported frequently in recent years.^6^, ^7^ Moreover, case series reports presented various treatment procedures and different results.

Concomitant driver gene mutations in *EGFR* and *ALK* have been detected in 1.3%‐15.4% of the patients with non‐small cell lung cancer (NSCLC), depending on the method used.^8^, ^9^ Among the patients harboring *EGFR*/*ALK* coalterations, some responded to treatment with an EGFR‐TKI (ie, gefitinib, erlotinib, icotinib, or afatinib),^8^, ^10^, ^11^, ^12^, ^13^ while others responded to treatment with an ALK‐TKI (crizotinib) 9, 13 or both.^14^, ^15^ To the best of our knowledge, there is currently no consensus for the optimal management for these patients. Although several studies provided recommendations on how to treat these patients,^8^, ^9^, ^15^ we still need further evidence to improve the efficacy of the therapy. While the co‐occurrence of driver gene mutations in patients harboring a *ROS1* rearrangement has been described as rare,^16^, ^17^, ^18^ Wiesweg et al recently reported that the rate of concomitant mutations could be as high as 36%.^19^ They detected *ROS1*/*ALK*, *ROS1*/*EGFR*, and *ROS1*/*KRAS* coalterations, and accordingly, new studies are required to establish how to treat these patients.

KRAS proto‐oncogene (*KRAS*) and B‐Raf proto‐oncogene (*BRAF*) have been frequently studied in NSCLC, and mutation testing for these genes is recommended in the National Comprehensive Cancer Network guidelines for NSCLC *KRAS* mutations have been found more frequently in non‐Asian patients and former or current smokers and have been associated with mucinous adenocarcinoma.^20^ Furthermore, evidence suggested that *KRAS* mutations combined with *EGFR* mutations or *ALK* rearrangements could negatively impact the effects of TKI therapy.^6^, ^13^, ^21^, ^22^ Meanwhile, the *BRAF* V600E mutation has been reported to be mutually exclusive with *EGFR* and *KRAS* mutations 23 but has been found to co‐occur with other driver gene mutations.^19^

Rare driver gene mutations, such as the erb‐b2 receptor tyrosine kinase 2 (*HER2*) mutation or MET proto‐oncogene (*MET*) exon 14 skipping, have also been found to co‐occur with other oncogenic driver mutations and were reported to respond well to TKI therapy.^12^, ^24^ Meanwhile, other concomitant mutations, such as *TP53* or *PIK3CA* mutations combined with *EGFR* mutations or *ALK* rearrangements, have been found to influence the treatment effect of TKI therapy.^25^, ^26^, ^27^, ^28^ However, mutation testing for these genes is not routinely performed in most clinical practices. Since there is no consensus for treating patients with concomitant driver gene mutations, studying coalterations in *EGFR*, *ALK*, *ROS1*, *KRAS*, and *BRAF* remains vital to improve treatments for these patients. Therefore, in this center, alterations in these five driver genes were regularly tested at diagnosis, and the clinical features and outcomes were analyzed for the patients with concomitant mutations.

## 2 PATIENTS AND METHODS

### 2.1 Patients

Between June 2014 and June 2017, 3774 samples from stage IIIB or IV Chinese patients diagnosed with NSCLC were screened for the five driver gene mutations. All patients provided written informed consent before molecular detection. All patients with concomitant mutations who received therapy at the center were included in the study. The EGFR‐TKIs were gefitinib, erlotinib, icotinib, or afatinib, the ALK‐TKIs were crizotinib or alectinib, and the ROS1‐TKI was crizotinib. All the patients received TKI doses consistent with the package insert recommendations. Follow‐up data were collected until July 2018 to assess progression‐free survival (PFS) and overall survival (OS). This study was approved by the Ethics Committee of Shanghai Pulmonary Hospital (2014‐FK02).

### 2.2 ARMS for the detection of *EGFR*, *ALK*, *ROS1*, *KRAS*, and *BRAF* mutations

Formalin‐fixed paraffin‐embedded (FFPE), fine/core needle aspiration, biopsy, or pleural effusion samples were used for the detection of alterations in the *EGFR*, *KRAS*, *BRAF*, *ALK*, and *ROS1* genes. Genomic DNA and total RNA were extracted from FFPE samples using the AmoyDx FFPE DNA/RNA extraction kit (ADx‐FF03, Amoy Diagnostics, Xiamen, China) according to the manufacturer's recommendations, and from all other samples using the AmoyDx Tissue DNA/RNA extraction kit (ADx‐TI03, Amoy Diagnostics). *EGFR*, *KRAS*, and *BRAF* mutations were detected using 80 ng, 120 ng, and 10 ng of DNA, respectively. *ALK* and *ROS1* rearrangements were detected using 100‐1000 ng of total RNA. The *EGFR* 29 Mutations Detection Kit (ADx‐EG01), *KRAS* Mutation Detection Kit (ADx‐KR01), *BRAF* V600 Mutations Detection Kit (ADx‐BR01), *EML4‐ALK* Fusion Gene Detection Kit (ADx‐AE01), and *ROS1* Gene Fusions Detection Kit (ADx‐RO02) from Amoy Diagnostics were used to detect alterations. For *ALK* and *ROS1* reverse transcription, RNA and 0.5 μL of EA Reverse Transcriptase were added to an EA RT and ROS1 RT Reaction Mix tube, respectively. The samples were then incubated for 1 hour at 42℃ followed by 5 minutes at 95℃. All real‐time qPCRs were performed on a Stratagene Mx3000P^™^ cycler (Agilent, Santa Clara, CA, USA) using the following program: 5 minutes at 95℃ (1 cycle); 25 seconds at 95℃, 20 seconds at 64℃, and 20 seconds at 72℃ (15 cycles); 25 seconds at 93℃, 35 seconds at 60℃, and 20 seconds at 72℃ (31 cycles). Ultrapure water was used as negative control, different commercial products were used as positive control for different gene detection, and the conservative sequences of the corresponding gene were used as quality control. Alterations in *EGFR*, *ALK*/*ROS1*, *KRAS*, and *BRAF* were defined as Ct values <26, <30, <26, and <28, respectively. For samples positive for *ALK* and *ROS1* rearrangements, DNA sequencing was performed to distinguish the variants as previously described.^29^, ^30^

### 2.3 Statistical analysis

All statistical analyses were performed using the SPSS v.20 software (SPSS Inc, Chicago, IL, USA). Comparisons of clinicopathological features in Table 1 and 2 were all evaluated using Pearson Chi‐square test, except that the analysis of pathology was evaluated using Fisher's exact test in Table 2. PFS and OS were analyzed using the Kaplan‐Meier method. The two‐sided significance level was set at *P* < 0.05.

**Table 1:** Clinicopathological features of *EGFR*, *ALK*, and *ROS1* single mutations with their concomitant mutations

	*EGFR*			*ALK*			*ROS1*		
	Concomitant n(%)	Single n(%)	*P*	Concomitant n(%)	Single n(%)	*P*	Concomitant n(%)	Single n(%)	*P*
Sex									
Female	25 (58.1)	61 (61.0)	0.853	11 (55.0)	51 (51.0)	0.810	1 (12.5)	30 (60.0)	0.020
Male	18 (41.9)	39 (39.0)		9 (45.0)	49 (49.0)		7 (87.5)	20 (40.0)	
Age (y)									
˂65	29 (67.4)	59 (59.0)	0.357	15 (75.0)	84 (84.0)	0.521	6 (75.0)	40 (80.0)	>0.9999
≥65	14 (32.6)	41 (41.0)		5 (25.0)	16 (16.0)		2 (25.0)	10 (20.0)	
Smoking status									
Never/light	34 (79.1)	77 (77.0)	0.831	14 (70.0)	73 (73.0)	>0.9999	5 (62.5)	38 (76.0)	0.666
Smoking	9 (20.9)	23 (23.0)		6 (30.0)	27 (27.0)		3 (37.5)	12 (24.0)	
Pathology									
Adenocarcinoma	39 (90.7)	91 (91.0)	>0.9999	20 (100.0)	90 (90.0)	0.210	7 (87.5)	42 (84.0)	>0.9999
Others	4 (9.3)	9 (9.0)		0(0)	10 (10.0)		1 (12.5)	8 (16.0)	

**Table 2:** Clinicopathological features of *EGFR* concomitant or single mutation patients treated with first‐line EGFR‐TKI

	Concomitant n(%)	Single n(%)	*P*
Sex			
Female	9 (81.8)	61 (61.0)	0.208
Male	2 (18.2)	39 (39.0)	
Age			
˂65	7 (63.6)	59 (59.0)	>0.9999
≥65	4 (36.4)	41 (41.0)	
Smoking status			
Never/light	10 (90.9)	77 (77.0)	0.451
Smoking	1 (9.1)	23 (23.0)	
Pathology			
Adenocarcinoma	11 (100.0)	91 (91.0)	0.595
Others	0	9 (9.0)	
Treatment effect			
PR	7 (63.6)	66	0.613
SD	2 (18.2)	26	
PD	2 (18.2)	8	

## 3 RESULTS

### 3.1 Frequency and outcomes of patients harboring concomitant mutations

**Figure 1:**
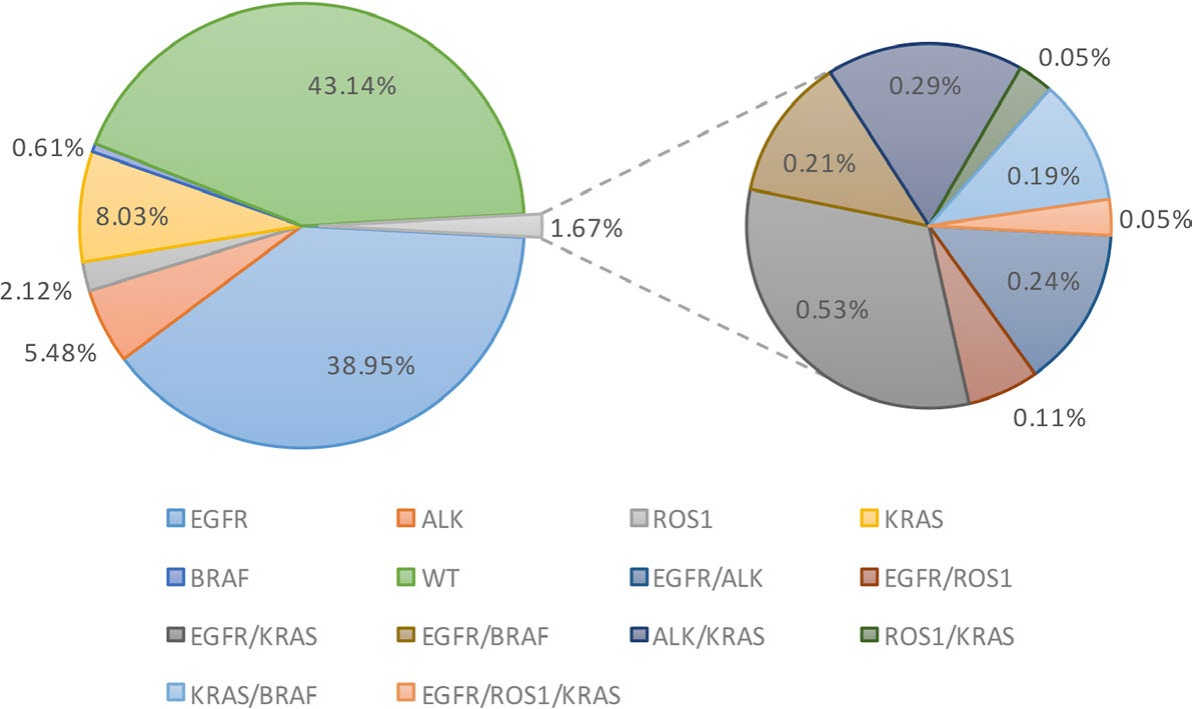
Frequency of *EGFR*, *ALK*, *ROS1*, *KRAS* and *BRAF* mutations in NSCLC patients. Wild‐type of the five genes was 43.14%, concomitant mutations was 1.67% and *EGFR*/*KRAS* was the most frequent mutation type (0.53%). Other mutation types are also listed in the figure

Among the 3774 patients tested, the single *EGFR*, *ALK*, *ROS1*, *KRAS*, and *BRAF* mutation rates were 39.0% (1470), 5.5% (207), 2.1% (80), 8.0% (303), and 0.6% (23), respectively. Sixty‐three patients (1.7%) harbored mutations in two or three of these genes (Figure 1), and among these patients, *EGFR/KRAS* was the most frequent coalteration (31.7%), followed by *ALK/KRAS* (17.5%). Out of 3774 patients, only two harbored a triple *EGFR/ROS1/KRAS* coalteration. Within the patients harboring coalterations with an *EGFR*, *ALK*, or *ROS1* alteration, 21 received TKI therapy as first‐line treatment, with an objective response rate (ORR) of 61.9% (13/21) and a median PFS of 11.8 months.

### 3.2 Patients harboring a coalteration with an *EGFR* mutation

**Figure 2:**
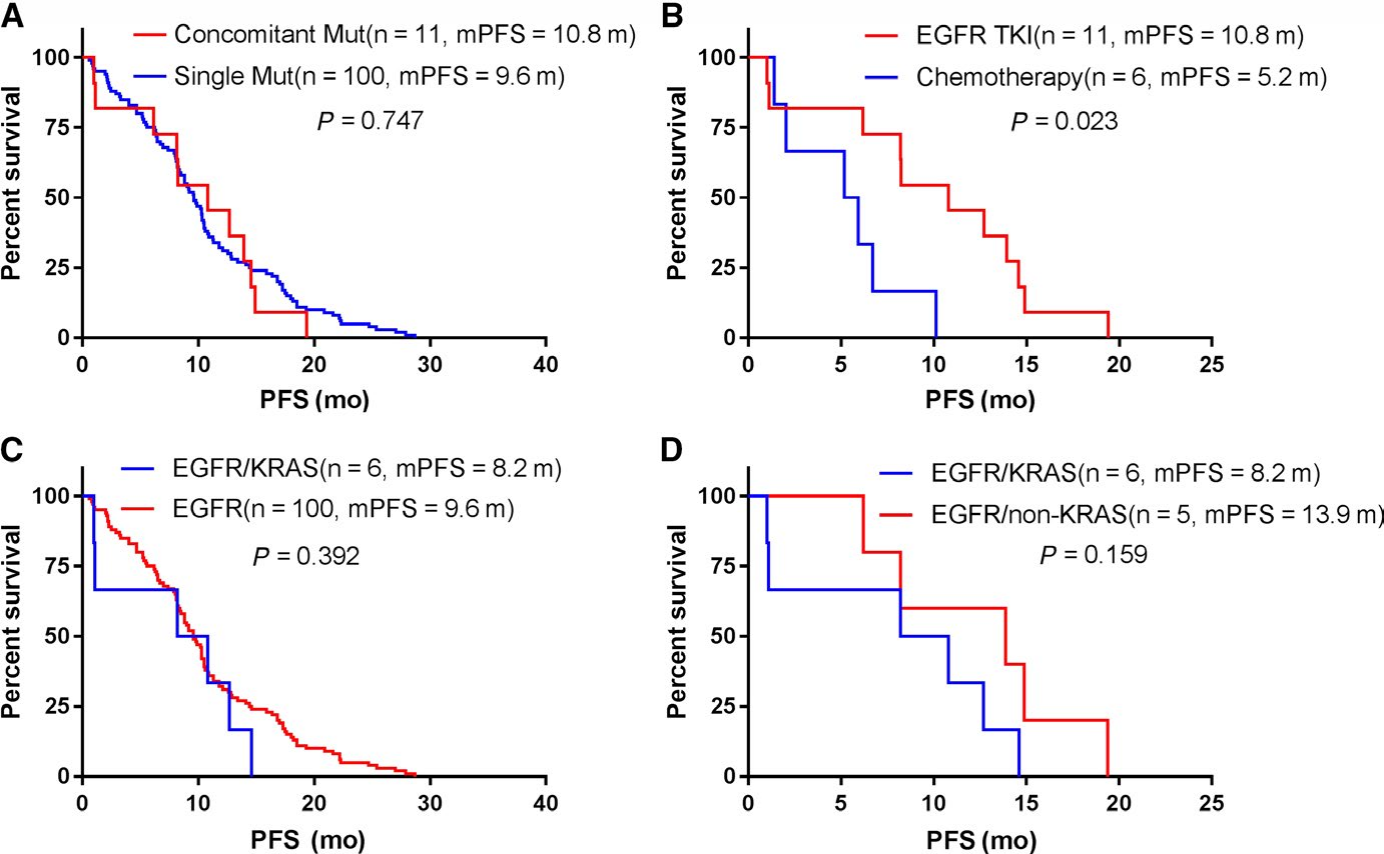
Clinical outcomes of *EGFR* concomitant mutation patients. Survival data were analyzed using Kaplan‐Meier method. A, PFS between *EGFR* concomitant mutation and single *EGFR* mutation patients treated with first‐line TKIs. B, PFS of *EGFR* concomitant mutation patients treated with first‐line TKI or chemotherapy. C, PFS between *EGFR/KRAS* concomitant and *EGFR* single mutation patients treated with first‐line TKIs. D, PFS of *EGFR/KRAS* and *EGFR*/non‐*KRAS* concomitant mutation patients treated with first‐line TKIs

Out of 1513 patients harboring an *EGFR* mutation, 43 (2.8%) carried an additional alteration. Notably, among the patients harboring an *EGFR* 19DEL or L858R mutation, 15 received EGFR‐TKI therapy as first‐line treatment and had a tumor response assessment (10 partial responses [PR], three stable diseases [SD], and two progressive diseases [PD]) with 11 patients achieving PFS, while seven received chemotherapy and had a tumor response assessment (two PR, three SD, and two PD) with six patients achieving PFS. We compared the clinical features of 100 randomly selected patients harboring a single *EGFR* mutation and receiving EGFR‐TKI therapy as first‐line treatment with those of the 43 patients harboring coalterations with an *EGFR* mutation but found no significant differences (Table 1). Furthermore, when we compared the clinical features and treatment effect of the 11 patients of the PFS group to those of the 100 randomly selected patients, we found no statistically significant differences (Table 2). Moreover, the PFS between the patients harboring a single *EGFR* mutation and those harboring coalterations with an *EGFR* mutation were not significantly different (10.8 vs 9.6 months, *P* = 0.747, Figure 2A). We also explored whether the patients harboring co‐alterations with an *EGFR* mutation benefited from TKI therapy or chemotherapy. The results showed that patients receiving TKI therapy as first‐line treatment (11 patients with coalterations: three *EGFR/ALK*, five *EGFR/KRAS*, two *EGFR/BRAF*, and one *EGFR/ROS1/KRAS*) had improved PFS compared to those receiving chemotherapy (six patients with coalterations: two *EGFR/ALK*, three *EGFR/KRAS*, and one *EGFR/ROS1/KRAS*) (10.8 vs 5.2 months, *P* = 0.023, Figure 2B). In contrast, sex, age, and smoking status were not associated with PFS of first‐line treatment (Table 3). For the patients harboring *EGFR/KRAS* and *EGFR*/non‐*KRAS* coalterations, and receiving EGFR‐TKI therapy as first‐line treatment, the ORR was 62.5% (5/8) and 71.4% (5/7), respectively. Finally, the PFS comparisons between patients harboring an *EGFR/KRAS* coalteration and those harboring a single *EGFR* mutation (Figure 2C), or between patients harboring an *EGFR/KRAS* coalteration and those harboring an *EGFR*/non‐*KRAS* coalteration (Figure 2D) showed no significant differences (*P* = 0.392 and *P* = 0.159, respectively).

**Table 3:** Survival analysis of *EGFR* concomitant mutation patients treated with first‐line EGFR‐TKI or chemotherapy

	No.(N = 17)	*P*
Sex		
Female	12	0.456
Male	5	
Age (y)		
˂65	12	0.814
≥65	5	
Smoking status		
Never/light	15	0.799
Smoking	2	
Treatment		
TKIs	11	0.023
Chemotherapy	6	

### 3.3 Patients harboring a coalteration with an *ALK* rearrangement

Out of 227 patients harboring an *ALK* rearrangement, 20 (8.8%) carried an additional alteration. Eight of these patients received crizotinib, with an ORR of 37.5% (3/8) across all treatment lines and 60.0% (3/5) for first‐line treatment (Table 4). We compared the clinical features of 100 randomly selected patients harboring a single *ALK* rearrangement with those of the 20 patients harboring a coalteration with an *ALK* rearrangement but found no significant differences (Table 1). At the time of the study, only three of the patients who received crizotinib as first‐line treatment achieved PFS. Therefore, we did not compare the outcomes of these patients with those of the patients harboring a single *ALK* rearrangement.

**Table 4:** Characteristics of *ALK* and *ROS1* concomitant mutation patients

Patient No.	Sex	Age	Smoking history (pack year)	Pathology^a^	Stage	Mutation	ALK/ROS1 variant	First‐line treatment	Effect	PFS (mo)	ALK(ROS1)‐TKI treatment line	TKI Effect	TKI PFS (mo)
1	M	48	0	A	IV	EGFR/ALK	/	Chemotherapy	SD	10.1	/		
2	F	69	0	A	IIIA	EGFR/ALK	V1	Chemotherapy	PR	5.9	/		
3	F	46	0	A	IIIB	ALK/KRAS	V3	Crizotinib	PR	/	1	PR	
4	M	58	45	A	IV	ALK/KRAS	/	Crizotinib	PD	1.1	1	PD	1.1
5	M	60	10	A	IV	ALK/KRAS	V3	Crizotinib	PR	28.0	1	PR	28.0
6	M	36	5	A	IV	ALK/KRAS	V5	Crizotinib	PR	14.8	1	PR	14.8
7	M	55	25	A	IV	ALK/KRAS	V1	Chemotherapy	SD	/	/		
8	F	51	0	A	IV	ALK/KRAS	V3	Crizotinib	SD	/	1	SD	
9	F	54	0	A	IV	ALK/KRAS	/	Chemotherapy	SD	9.3	2	SD	
10	M	73	30	A	IV	ALK/KRAS	/	Chemotherapy	SD	83.2	3	PD	1.2
11	F	49	0	A	IV	ALK/KRAS	V2	Chemotherapy	PD	1.2	3	SD	2.9
12	M	59	80	A	IV	EGFR/ROS1	EZR‐E10;ROS1‐E34	Chemotherapy	SD	/	/		
13	M	54	0	A	IIIB	EGFR/ROS1	CD74‐E6;ROS1‐E34	Crizotinib	SD	/	1	SD	
14	M	53	0	A	IV	EGFR/ROS1/KRAS	EZR‐E10;ROS1‐E34	Chemotherapy	PD	2.0	2	PD	1.0
15	M	52	20	A	IV	EGFR/ROS1/KRAS	/	Gefitinib	PR	12.7	/		
16	M	77	0	A	IIIA	ROS1/KRAS	EZR‐E10;ROS1‐E34	Chemotherapy	PD	0.9	/		

a A, adenocarcinoma

### 3.4 Patients harboring a coalteration with a *ROS1* rearrangement

Out of 88 patients harboring an *ROS1* rearrangement, eight (9.1%) had coalterations. We compared the clinical features of 50 randomly selected patients harboring a single *ROS1* rearrangement with those of the eight patients harboring a coalteration with an *ROS1* rearrangement and found that coalterations with an *ROS1* rearrangement occurred more frequently in male patients (*P* = 0.020) (Table 1). Concerning the outcomes, among the two patients treated with crizotinib, one had SD after receiving crizotinib as first‐line treatment, and the other had PD after receiving crizotinib as second‐line treatment (Table 4). Furthermore, two patients harbored *EGFR/ROS1/KRAS* triple co‐alterations (patient 14 and 15). Patient 14 had PD after receiving crizotinib as second‐line treatment and PR after receiving icotinib as third‐line treatment (PFS of 27.5 months), whereas patient 15 had PR after receiving gefitinib as first‐line treatment (PFS of 12.7 months).

### 3.5 Patients harboring a coalteration with a *KRAS* or *BRAF* mutation

Out of the 42 patients harboring a coalteration with a *KRAS* mutation, 13 (31.0%) had a G12C substitution, 10 (23.8%) G12D, six (14.3%) G12S, five (11.9%) G12V, and eight (19.0%) a different mutation. Furthermore, three patients harbored two types of *KRAS* mutation with one each carrying G12R/C, G12D/V, and G12S/C mutations. Concerning the 22 patients harboring *EGFR/KRAS* coalterations, the most frequent types of *KRAS* mutation were G12C (31.8%) and G12D (31.8%). Meanwhile, G12C was the most frequent *KRAS* mutation (54.5%) among the 11 patients harboring *ALK/KRAS* coalterations. Seventeen of the 42 patients harboring a coalteration with a *KRAS* mutation received first‐line chemotherapy, with an ORR of 26.7% (four PR, five SD, and six PD) and a median PFS of 4 months. Moreover, 15 patients harbored a coalteration with a *BRAF* mutation, with eight and seven patients carrying *EGFR/BRAF* and *KRAS/BRAF* coalterations, respectively. Seven of these patients received EGFR‐TKI therapy as first‐line treatment (two PR and one SD), and two of them achieved PFS (19.4 and 14.9 months, respectively). In comparison, five patients received chemotherapy (four SD and one PD), and three of them achieved PFS (8.1, 2.8, and 17.4 months, respectively).

## 4 DISCUSSION

Concomitant driver gene mutations in NSCLC patients have been reported in previous case series studies. However, the standard treatment of these patients was still ongoing. Therefore, it is of great importance to determine the clinical features and outcomes of these patients to provide the most effective treatments. To the best of our knowledge, this report is the first comprehensive study of concomitant driver gene mutations in Chinese patients harboring *EGFR*, *ALK*, *ROS1*, *KRAS*, and *BRAF* alterations. We identified 63 patients who harbored concomitant mutations. Our data showed that patients harboring coalterations with an *EGFR* mutation had better PFS with TKI therapy than with chemotherapy, while coalterations with an *ROS1* rearrangement occurred more frequently in male patients.

A previous study reported that concomitant mutations were found in approximately 5% of the patients with lung adenocarcinoma.^31^ However, using more precise detection methods, the same patient cohort could exhibit different results.^9^ Indeed, *EGFR/ALK* coalterations have been reported to occur in 3.9%‐13.6% and 15.4%‐18.8% of the patients harboring *EGFR* mutations and *ALK* rearrangements, respectively.^8^, ^9^, ^32^ Furthermore, *EGFR/KRAS* coalterations have been reported to occur in 5.8%‐35.8% of the patients harboring *EGFR* mutations.^16^, ^32^ In the present study, *EGFR/ALK* coalterations were detected in 0.6% and 4.0% of the patients harboring *EGFR* and *ALK* alterations, respectively, which was lower than in other available studies. Most of the samples we used had been prepared for cytological analyses, which could explain why certain mutation‐positive tumor cells were not detected by comparison with detection performed on resected tumor tissues or biopsy tissue samples. Besides, the use of more precise methods to detect the alterations, such as next‐generation sequencing (NGS), would most likely result in a higher ratio of concomitant mutations.

The choice between EGFR‐TKI and ALK‐TKI therapy as first‐line treatment for patients harboring *EGFR/ALK* concomitant mutations has been debated since their discovery. In certain studies, EGFR‐TKI therapy gave better results, while in others it was the other way around.^8^, ^9^, ^10^, ^11^, ^14^, ^15^ Apart from the influence of the level of protein phosphorylation on treatment effect, we think that there may be at least three other possible explanations for these contradicting observations. First, we must consider tumor heterogeneity. Different mutations may coexist in the same tumor cells 15 or may be present in different areas (ie, different cells) of the tumor.^33^ If tumor cells carry both *EGFR* and *ALK* alterations, the two types of inhibitors may be able to kill tumor cells and could have a positive treatment effect. However, in cases where the mutations affect different tumor cells, a given type of TKI can only target corresponding tumor cells (eg, EGFR‐TKI can only target tumor cells with EGFR alterations), and other tumor cells (ie, not carrying the targeted mutation) may be able to proliferate rapidly. Second, the existence of gene mutation subtypes may impact the treatment effect. For example, various *ALK* rearrangement variants result in different responses to ALK‐TKI therapy and resistance patterns.^34^, ^35^ Furthermore, a similar phenomenon has also been reported for classical *EGFR* mutations.^36^, ^37^ In this situation, the types of mutation should be considered carefully before making any decision regarding the treatment. Finally, we cannot exclude the possibility of unknown mechanisms impacting the outcomes. The pathological subtype, passenger mutations, and mutations in other genes,^27^, ^28^ as well as smoking‐related genomic patterns,^38^ might influence the treatment effect. In this study, since only three of the patients harboring *EGFR/ALK* coalterations and receiving TKI therapy achieved PFS, we did not compare the clinical outcomes of these patients with those of the patients harboring a single *EGFR* or *ALK* alteration. Regarding the whole group of patients harboring coalterations with an *EGFR* mutation, we did not record significant differences with the group of patients harboring a single *EGFR* mutation following TKI therapy. However, they benefited more from TKI therapy than from chemotherapy. In previous studies, *KRAS* mutations have been associated with primary resistance to EGFR‐TKI therapy.^6^, ^21^, ^22^ In this study, we did not record significant differences of PFS between the patients harboring *EGFR/KRAS* coalterations and those harboring a single *EGFR* mutation, or between the patients harboring *EGFR/KRAS* coalterations and those harboring *EGFR*/non‐*KRAS* coalterations. However, the study included only a small number of patients with such coalterations, and future studies would greatly benefit from larger patient cohorts.

In this study, the cohort of patients harboring coalterations with an *ALK* or *ROS1* rearrangement who received TKI therapy as first‐line treatment was too small to compare the clinical outcomes with those of patients harboring a single *ALK* or *ROS1* rearrangement. Nevertheless, we were able to determine that ALK‐TKI therapy for the treatment of patients harboring a coalteration with an *ALK* rearrangement was more efficient in first‐line treatment than in later lines of treatment. Moreover, for patients harboring *EGFR/ROS1/KRAS* triple coalterations, EGFR‐TKI therapy may have been more efficient than ROS1‐TKI therapy. However, this result must be supported by additional evidence.

*KRAS* mutations were reported to be negatively correlated with the treatment effect of TKI therapy, which highlighted the lack of effective targeted drugs for these patients. Therefore, chemotherapy remains the primary therapy for patients harboring *KRAS* mutations. However, we found that the PFS for chemotherapy as first‐line treatment for patients harboring coalterations with a *KRAS* mutation was only four months. For patients with single *KRAS* mutation, G12C, G12D, and G12V have been reported as the most frequent mutation subtypes.^39^ For patients harboring concomitant mutations, G12C/D and G12C *KRAS* mutation subtypes were the most frequent in *KRAS/EGFR* and *KRAS/ALK* coalterations, respectively. The development of targeted therapy for the treatment of patients harboring the *BRAF* V600E mutation has also been relatively slow until the combination of dabrafenib plus trametinib was approved for the treatment of these patients.^40^, ^41^ However, no patient included in this study received this combination therapy. Moreover, our data suggested that the PFS of patients harboring *EGFR/BRAF* coalterations could likely be improved with the use of TKI therapy.

Rare mutations, such as the *HER2* mutation and *MET* exon 14 skipping, can co‐occur with *EGFR* mutations, and patients with these types of coalteration have been reported to respond well to EGFR‐TKI or combined therapy.^12^, ^24^ In our center, patients first diagnosed with NSCLC were routinely screened for *EGFR*, *ALK*, *ROS1*, *KRAS*, and *BRAF* alterations. Notably, *HER2* mutations, *MET* exon 14 skipping, and *RET* rearrangements were only detected in patients who carried wild‐type alleles of *EGFR*, *ALK*, *ROS1*, *KRAS*, and *BRAF*. As mentioned before, NGS can be used to detect mutations that would otherwise be missed by regular screening methods. Besides coalterations among the five driver genes studied here, mutations in *TP53*, *PIK3CA*, and other genes can also co‐occur with *EGFR* or *ALK* alterations, in which case they have been shown to decrease the treatment effect of TKI therapy.^25^, ^26^, ^27^, ^28^ For patients affected by this type of coalteration, combination therapy such as TKI plus chemotherapy should be considered. However, to date, these mutations have not been routinely tested in most clinical practices. Collectively, these data highlight the importance and necessity of studying coalterations involving one or more of the *EGFR*, *ALK*, *ROS1*, *KRAS*, and *BRAF* driver genes.

In conclusion, concomitant driver gene mutations define a small group of NSCLC patients. Patients harboring coalterations with an *EGFR* mutation tend to benefit more from TKI therapy than from chemotherapy. However, further studies are needed to evaluate the treatment outcomes of patients harboring an *ALK* or *ROS1* rearrangement combined with an *EGFR* mutation.

## CONFLICT OF INTEREST

The authors declare that they have no competing interests.

## DATA AVAILABILITY STATEMENT

*Cancer Medicine* encourages authors to share the data and other artefacts supporting the results in the paper by archiving it in an appropriate public repository. Authors may provide a data availability statement, including a link to the repository they have used, in order that this statement can be published in their paper. Shared data should be cited.
